# Anti-methicillin-resistant *Staphylococcus aureus* activity and safety evaluation of 14-O-[(5-ethoxycarbonyl-4,6-dimethylpyrimidine-2-yl) thioacetyl] mutilin (EDT)

**DOI:** 10.1038/s41598-023-42621-0

**Published:** 2023-09-14

**Authors:** Yuhang Zhou, Yunpeng Yi, Jing Yang, Hongjuan Zhang, Qinqin Liu, Shengyi Wang, Wanxia Pu, Ruofeng Shang

**Affiliations:** 1 Key Laboratory of New Animal Drug Project, Gansu Province/Key Laboratory of Veterinary Pharmaceutical Development, Ministry of Agriculture and Rural Affairs/Lanzhou Institute of Husbandry and Pharmaceutical Sciences of CAAS, No. 335, Qilihe District, Lanzhou, 730050 People’s Republic of China; 2 Shandong Provincial Animal and Poultry Green Health Products Creation Engineering Laboratory, Institute of Poultry Science, Shandong Academy of Agricultural Science, Jinan, 250023 People’s Republic of China; 3 Gansu Analysis and Research Center, Lanzhou, 730000 People’s Republic of China

**Keywords:** Drug safety, Pharmaceutics, Target validation

## Abstract

Infections caused by methicillin-resistant *Staphylococcus aureus* (MRSA) have threated the public health worldwide, which emphasizes the urgent need for new drugs with novel mechanism of actions. 14-O-[(5-ethoxycarbonyl-4,6-dimethylpyrimidine-2-yl) thioacetyl] mutilin (EDT) is a pleuromutilin compound with high activity against several Gram-positive bacteria in vitro and in vivo. This study aimed to verifying the potential anti-MRSA activity and evaluating the safety of EDT. In in vitro antibacterial activity assays, EDT exhibited potent antibacterial activity against MRSA isolated from clinic (minimum inhibitory concentration = 0.0313–0.125 μg/mL), increased post-antibiotic effect (PAE) values and limited potential for the development of resistance. Docking model and green fluorescent protein (GFP) inhibition assay further elucidated the higher antibacterial activities of EDT via mechanism of action. In safety evaluation, EDT exhibited low cytotoxic effect and acute oral toxicity in mice and avoided to significantly increase the number of revertant colonies of six tested strains in the Ames study. Furthermore, EDT displayed a moderate inhibitory effect on CYP3A4 and moderate stability in mouse and human liver microsomes, providing a promising agent for the development of new antimicrobial candidate.

## Introduction

The discovery and application of antibiotics have helped humans to fight bacterial infections to a great extent, making humans face the problem of infection from helpless to leisurely^1^. However, the emergence of drug resistance has led to many marketed antibacterial drugs reduced or lost effective. Unfortunately, the development speed of new antibiotics fails to keep up with the outbreak of resistant bacteria. Therefore, it is urgent to design and develop new molecular entity with novel antibacterial mechanism to solve this problem^2,3^.

**Figure 1:**
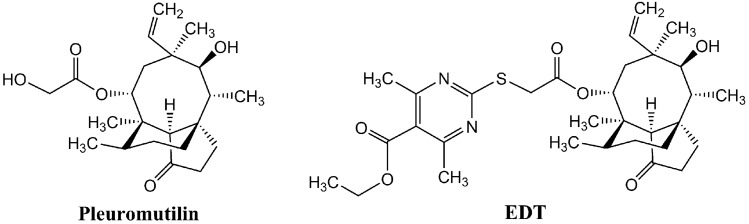
Chemical structure formulas of pleuromutilin and EDT.

Pleuromutilin (Fig. 1) was discovered and isolated from *Pleurotus mutilus* and *P. passeckerianu* as a diterpene natural product in 1951^4^. Because of its unusual tricyclic structure which can bind to the 50S ribosomal subunit of bacteria and disturb the protein synthesis, pleuromutilin show good antibacterial effects on Gram-positive bacteria and Mycoplasma^5–9^. The unique antibacterial mechanism of pleuromutilin has stimulated many researchers to modify it to tackle the multidrug resistant bacterial infections^9,10^, and thus the semisynthetic derivatives tiamulin, valnemulin, retapamulin and lefamulin were discovered and approved as antibiotics for marketing^11,14^.

It was reported that linking an aromatic heterocycle with polar groups to the C-14 side chain of pleuromutilin could significantly improve their antibacterial activities^15,16^. Inspired by this point, a variety of pleuromutilin derivatives bearing heterocyclic rings, such as oxadiazole, triazole and pyrimidine, were designed and synthesized for screening more efficacious drug candidates^16–20^. In our previous research, a novel pleuromutilin compound, 14-O-[(5-Ethoxycarbonyl-4,6-dimethylpyrimidine-2-yl) thioacetyl] mutilin (EDT, Fig. 1) with a substituted pyrimidine side chain, was synthesized, screened and proposed to be developed as a new drug for veterinary use. This compound displayed high activity against *Staphylococcus aureus* (*S. aureus*), methicillin-resistant *Staphylococcus aureus* (MRSA), methicillin-resistant *staphylococcus epidermidis* (MRSE) and *Bacillus subtilis *in vitro and in vivo^21^. For further evaluating the antibacterial activity of EDT, we tested its efficacy on clinical isolates of MRSA, post-antibiotic effect (PAE) and the development of resistance, as well as docking study and green fluorescent protein (GFP) inhibition assay. Furthermore, we conducted cytotoxicity, acute toxicity test, CYP450 effect, Ames test to assess its safety.

## Results

### In vitro anti-MRSA activity of EDT

**Figure 2:**
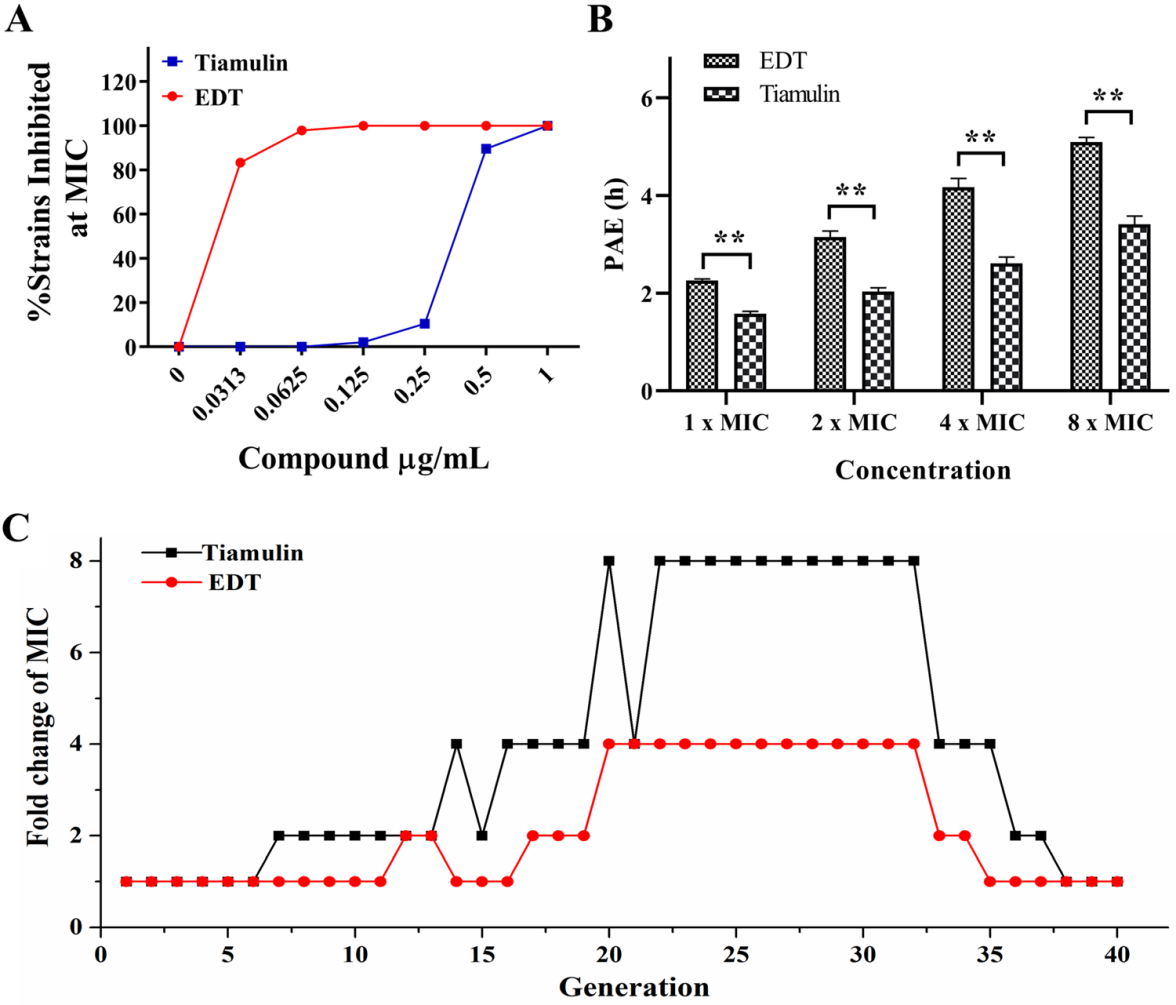
In vitro antibacterial activity of EDT and tiamulin fumarate. (**A**) Inhibition rate against clinical isolates of S. aureus; (**B**) PAE values; (**C**) development of MRSA resistance after repetitive treatments for 30 days. *P < 0.05, **P < 0.01.

We used a panel of clinical isolates of MRSA (n = 48) to assess their susceptibility to EDT. Tiamulin fumarate was chosen as a comparator because it was widely used as a clinical veterinary medicine. Minimum inhibitory concentration (MIC) values of EDT and tiamulin against 48 stains were 0.0313–0.125 μg/mL and 0.125–1 µg/mL, respectively, with slight fluctuation (MIC data were showed in Table S1). At the 0.0625 µg/mL, EDT inhibited 97.92% strains, much higher than that of tiamulin (0%). These results displayed that EDT showed higher antibacterial activity than that of tiamulin within the collections of clinical isolates (Fig. 2A).

Postantibiotic effect (PAE) is an important indicator for the evaluation of antimicrobial drugs. After removing drugs, the concentrations (Log CFU/mL) of the strains taken at 0, 1, 2, 3, 4, 5, 6, 8, 12, 18 and 24 h were showed in Fig. S2. Both of EDT and tiamulin showed increased PAE values in a concentration-dependent manner (Fig. 2B). At four tested concentrations, PAE values of EDT were all extremely significantly higher than that of tiamulin, suggesting that EDT displayed a better post-antibiotic response.

Resistance development of bacteria to a new chemical is an important indicator to design and evaluate it properties^22^. To evaluate the spontaneous resistance to EDT, we performed an induced resistance test at sublethal concentrations against MRSA for 40 passages (MIC data were showed in Table S2). After 7 generations of continuous culture of MRSA, MIC values of tiamulin showed twofold increase. At 22 generations, tiamulin induced bacterial resistance rapidly, displaying eightfold increase in the MIC values. By contrast, only fourfold increase was found in the MIC values of EDT. After removing drugs and culturing for additional 10 generations, MRSA recovered the initial sensitivity to EDT and tiamulin (Fig. 2C; Table S2).

### Comparison of antibacterial activities to tiamulin via mechanism of action

**Figure 3:**
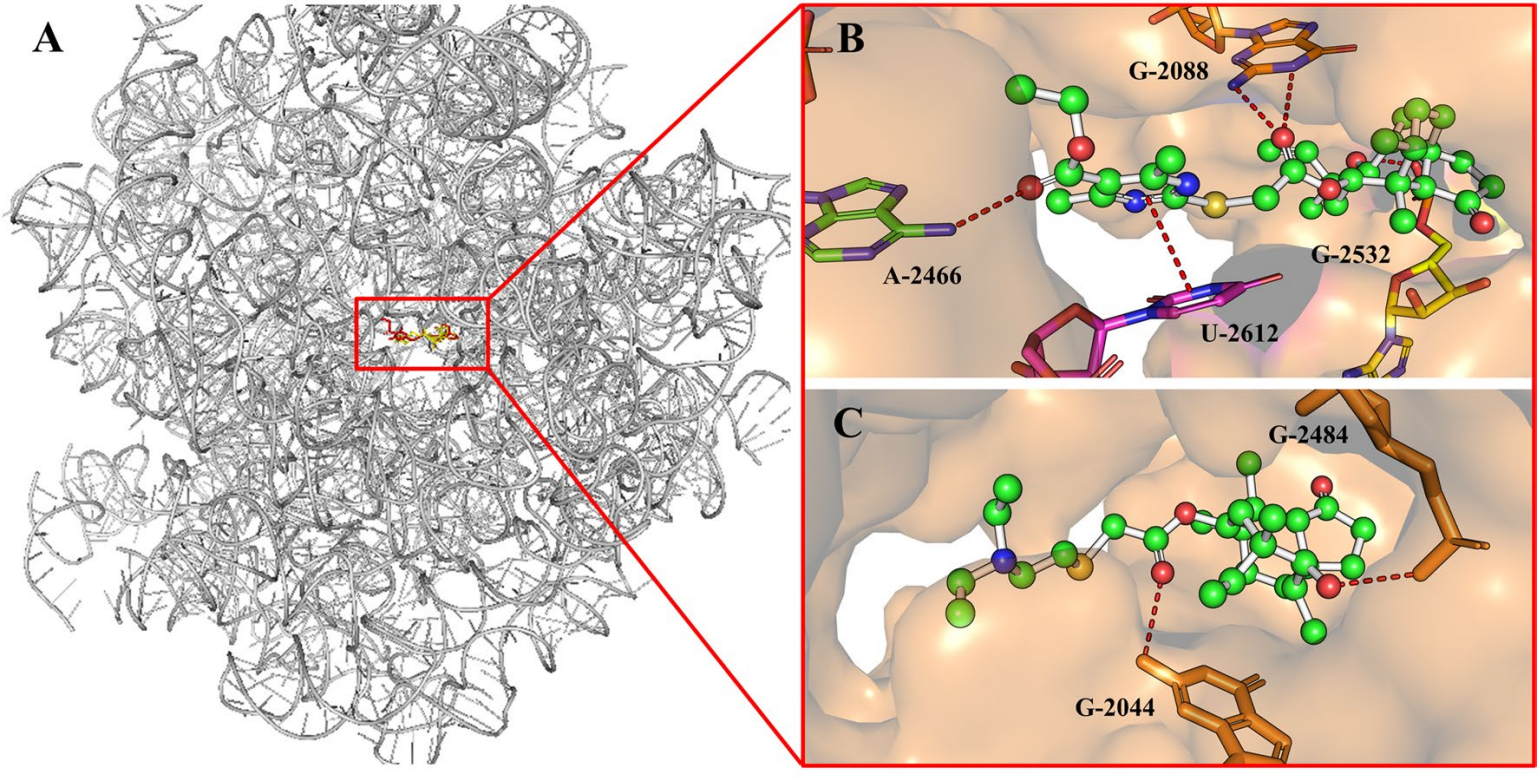
Superimposition of tiamulin (yellow) and the best conformation of EDT (red) docked to the binding pocket of ribosome (**A**). Docking mode of EDT (**B**) and tiamulin (**C**) into 1XBP.

Given the higher in vitro antibacterial activity of EDT, we performed molecular docking for further understanding its possible interaction modes with *D. radiodurans* 50S ribosome subunit. This study allowed us to potentially correlate the results from the antibacterial studies with the docking-based binding poses. Redocking of tiamulin into 1XBP placed the compound in the PTC as the X-ray structure. The optimal conformation of EDT presented a similar binding mode consistent with that of tiamulin within the binding site (Fig. 3A). The results revealed hydrogen bindings played the important role in the binding of EDT to 1XBP when flexible docked into the PTC (Fig. 3B). The side chain of EDT unfolded and the pyrimidine ring fragment was adjacent to the U-2612 residue forming a π-π bond. Four hydrogen bonds were formed between hydroxyl group and C=O (two esters) of EDT and the residue of G-2088 and A-2466. However, the redocked tiamulin formed two hydrogen bonds between hydroxyl group and C=O (esters) with G-2484 and G-2044 (Fig. 3C). Furthermore, the binding affinity of EDT (ΔG_b_ =  − 9.48 kcal/mol) was higher than that of tiamulin (ΔGb =  − 8.10 kcal/mol), which were in agreement with their antibacterial activities.

**Figure 4:**
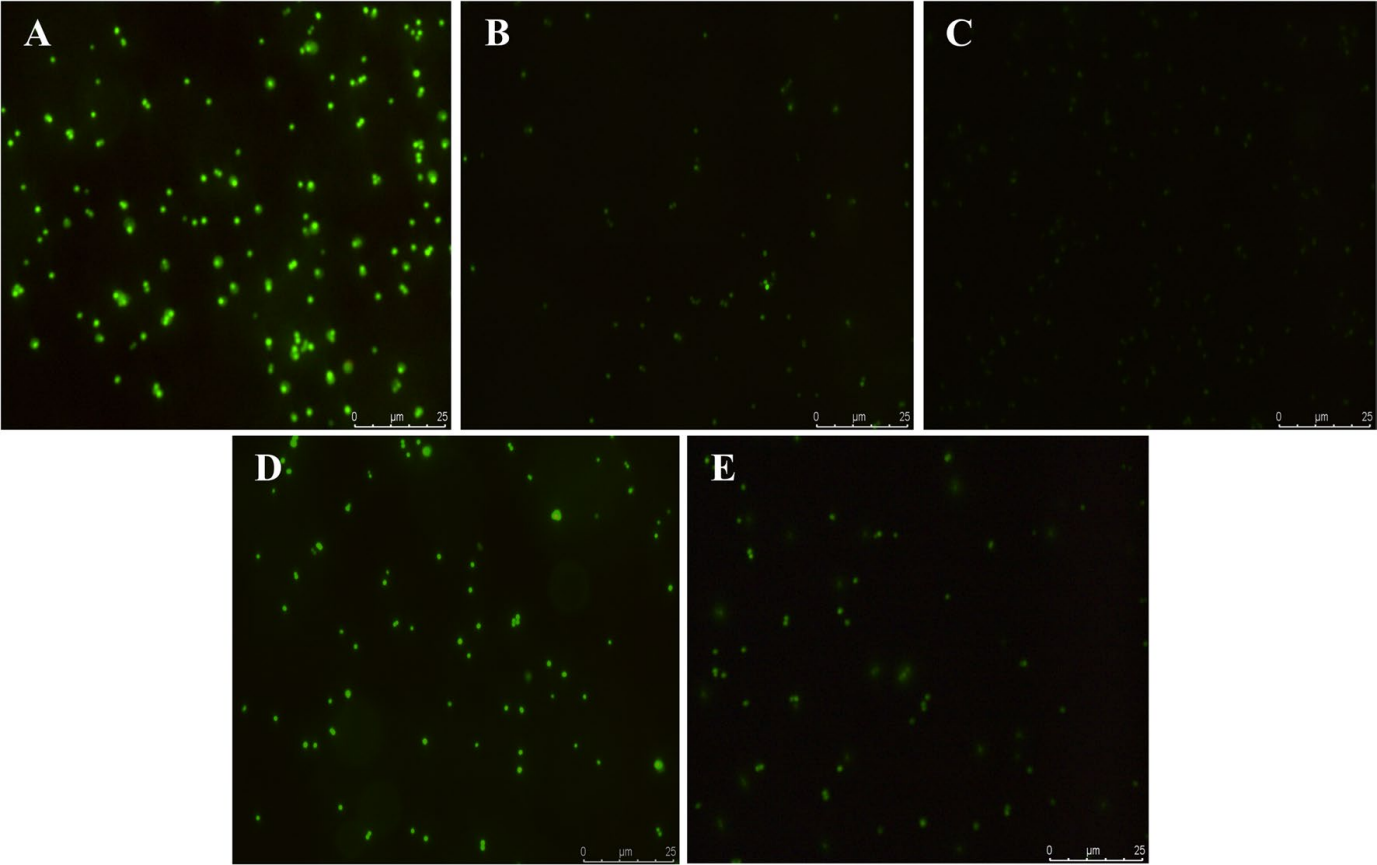
EDT and tiamulin inhibited GFP expression in *S. aureus* ATCC 29,213. (**A**) Control group, (**B**) 1 μM EDT, (**C**) 4 μM EDT, (**D**) 1 μM tiamulin, (**E**) 4 μM tiamulin. Scale bar: 25 μm.

To confirm the effect of EDT that was superior to that of tiamulin on protein synthesis, we used modified strains of *S. aureus* that ectopically expresses green fluorescent protein (GFP) until protein synthesis was hindered, resulting in the reduced the green fluorescence intensity. After co-incubating with the GFP stains for 4 h, EDT effectively inhibited the expression of GFP when compared to the control, which was easily determine from the reduced the green fluorescence intensity in Fig. 4. By comparison to tiamulin, the same dose of EDT (1 and 4 μM) effectively interfered with bacterial protein expression with reduced green fluorescence intensity.

### Safety evaluation

**Figure 5:**
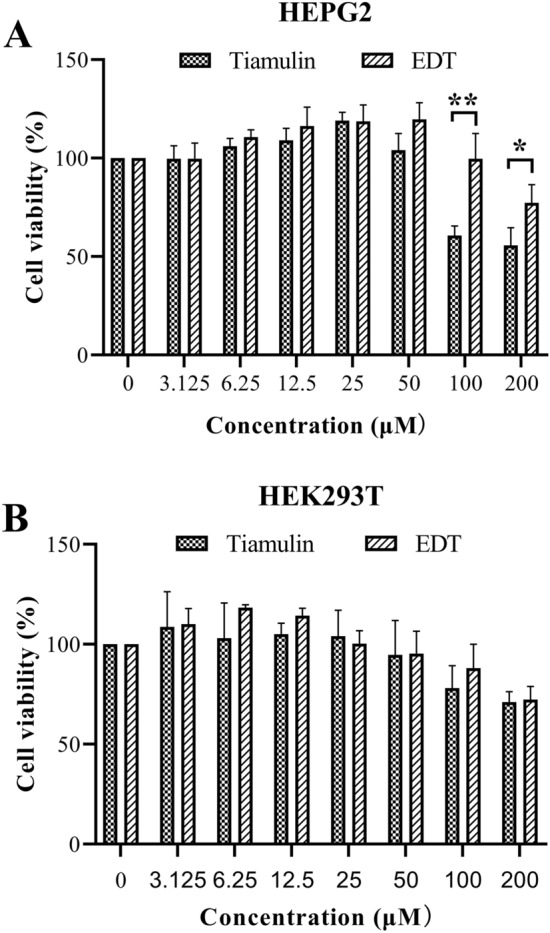
Cytotoxicity assay with EDT and tiamulin in the HEPG2 (**A**) and HEK293T (**B**). Each data bar was an average of three replicates. *P < 0.05, **P < 0.01.

Safety is one of the major concerns for an antibacterial agent in the development to clinically useful drug. Using HepG2 and HEK293T cells, preliminary cytotoxicity studies of EDT and tiamulin were performed through dose–response studies (Fig. 5). The cell viabilities of HepG2 induced by EDT at 100 and 200 μM were 99.68% and 77.46%, respectively, while the same dose of tiamulin eliminated more of the living cells, with the viability measured at 60.77% and 55.56%. However, for the cell viabilities of HEK293T, no significantly difference (*P* < 0.05) was observed between EDT and tiamulin at 100 and 200 μM, respectively.

**Figure 6:**
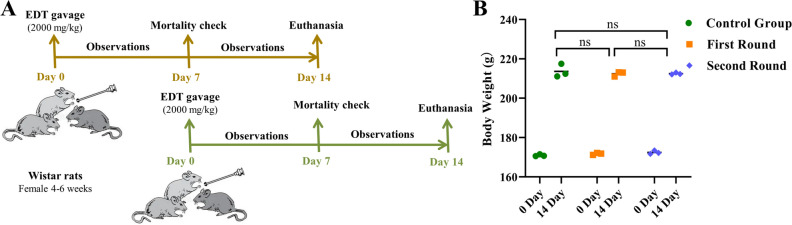
Experimental procedures for acute toxicity tests (**A**) and the changes of body weight (**B**).

After a single administration of 2000 mg/kg of EDT according to the OECD423 procedure^23^, animals did not show any significant changes or discomfort in treatment group during the 14 days of experiment (Fig. 6A). All animals survived until programmed euthanasia with ketamine hydrochloride. No difference food intake or water consumption was found when compared to that in the control group. Acute treatment with EDT did not produce alterations in body weight when compared to the control group (Fig. 6B). Macroscopic necropsy examination revealed no gross pathological lesions in any rats. The absolute and relative organ weights of heart, liver, spleen, lungs, kidneys, uterus and ovaries were summarized in Table 1, and no significant change (*P* > 0.05) in treatment groups was found when compared to that of the control group.

**Table 1:** Absolute and relative organ weights from female rats treated orally with a single dose of EDT.

Organ	Control group	First round treatment	Second round treatment	*P*_1_^a^	*P*_2_^b^
Absolute organ weight (g)					
Liver	9.78 ± 0.05	9.66 ± 0.17	9.69 ± 0.06	0.617	0.528
Kidneys	1.14 ± 0.04	1.11 ± 0.01	1.11 ± 0.03	0.692	0.736
Spleen	0.71 ± 0.03	0.70 ± 0.03	0.71 ± 0.03	0.944	1.000
Heart	0.72 ± 0.04	0.71 ± 0.06	0.75 ± 0.03	0.983	0.734
Lungs	1.42 ± 0.02	1.38 ± 0.03	1.40 ± 0.05	0.224	0.842
Ovaries	0.06 ± 0.00	0.06 ± 0.00	0.06 ± 0.00	0.384	0.455
Uterus	0.42 ± 0.01	0.45 ± 0.02	0.43 ± 0.03	0.079	0.760
Relative organ weight (%)					
Liver	4.58 ± 0.05	4.55 ± 0.06	4.56 ± 0.06	0.876	0.971
Kidneys	0.53 ± 0.01	0.52 ± 0.01	0.52 ± 0.01	0.625	0.737
Spleen	0.33 ± 0.01	0.33 ± 0.01	0.33 ± 0.01	0.984	0.994
Heart	0.34 ± 0.01	0.33 ± 0.03	0.35 ± 0.01	0.994	0.561
Lungs	0.67 ± 0.01	0.65 ± 0.02	0.66 ± 0.02	0.396	0.919
Ovaries	0.03 ± 0.00	0.03 ± 0.00	0.03 ± 0.00	0.443	0.428
Uterus	0.20 ± 0.01	0.21 ± 0.01	0.20 ± 0.01	0.093	0.731

^a^*P* values of significant differences between the control group and the first round treatment.

^b^*P* values of significant differences between the control group and the second round treatment.

Based on the results of the preliminary study, the maximum concentration of EDT was set to 2000 μg/plate. The results of the strains reverse mutation assay for EDT in the presence or absence of metabolic activation were shown in Table 2. Revertant colonies in all positive groups were more than twice of that induced by five concentrations of EDT and vehicle control. There was no significantly different between the number of revertant colonies induced by the vehicle control and EDT treatment groups. Furthermore, no dose-dependent increase in the EDT groups was observed for any of the strains, regardless of − S9 and + S9.

**Table 2:** Results of the Ames test of EDT.

Groups	S9	Experimental strains					
		TA97a	TA98	TA100	pKM101	TA102	TA1535
2000 μg/plate	+	44.00 ± 5.00	29.67 ± 2.33	137.00 ± 14.00	50.33 ± 3.67	236.33 ± 19.67	19.33 ± 3.67
	−	52.00 ± 6.00	32.33 ± 3.67	122.67 ± 11.33	47.00 ± 4.00	228.00 ± 16.00	17.67 ± 2.33
400 μg/plate	+	54.33 ± 4.67	25.00 ± 4.00	134.33 ± 12.67	53.00 ± 5.00	219.67 ± 18.33	26.00 ± 3.00
	−	56.00 ± 3.00	26.67 ± 3.33	129.67 ± 15.33	51.33 ± 3.67	211.00 ± 14.00	23.33 ± 2.67
80 μg/plate	+	50.33 ± 3.67	19.00 ± 3.00	118.67 ± 14.33	44.00 ± 4.00	223.67 ± 17.33	25.00 ± 4.00
	−	53.33 ± 5.67	23.33 ± 2.67	105.33 ± 13.67	41.33 ± 3.67	209.33 ± 13.67	23.33 ± 2.67
16 μg/plate	+	46.00 ± 4.00	24.00 ± 4.00	103.67 ± 9.33	54.00 ± 4.00	218.00 ± 9.00	27.00 ± 2.00
	−	44.33 ± 5.67	23.33 ± 2.67	101.00 ± 9.00	46.00 ± 5.00	205.00 ± 11.00	23.33 ± 1.67
3.2 μg/plate	+	57.67 ± 4.33	22.00 ± 2.00	119.67 ± 7.33	50.33 ± 2.67	214.33 ± 14.67	21.00 ± 4.00
	−	52.00 ± 3.00	21.67 ± 1.67	109.00 ± 8.00	42.00 ± 3.00	208.00 ± 15.00	19.67 ± 1.33
DMSO	+	57.33 ± 3.67	24.00 ± 2.00	131.33 ± 12.67	47.00 ± 4.00	212.00 ± 19.00	18.33 ± 2.67
	−	55.00 ± 4.00	21.33 ± 2.67	129.00 ± 13.00	43.33 ± 3.67	208.00 ± 17.00	17.00 ± 3.00
Positive control	+	1867.00 ± 89.00^a^	1080.67 ± 42.67^a^	582.00 ± 26.00^a^	782.33 ± 47.67^a^	979.00 ± 68.00^a^	740.00 ± 44.00^d^
	−	2472.00 ± 94.00^b^	972.00 ± 37.00^b^	1017.33 ± 39.67^c^	644.00 ± 38.00^c^	522.00 ± 45.00^b^	534.33 ± 38.67^e^

The units in groups were μg/plate. The used positive control chemicals were 2-aminofluorene (a), diaxone (b), methyl sulfonate (c), 2-aminoanthracene (d), and sodium azide (e).

### Inhibitory effects on cytochrome P450

**Figure 7:**
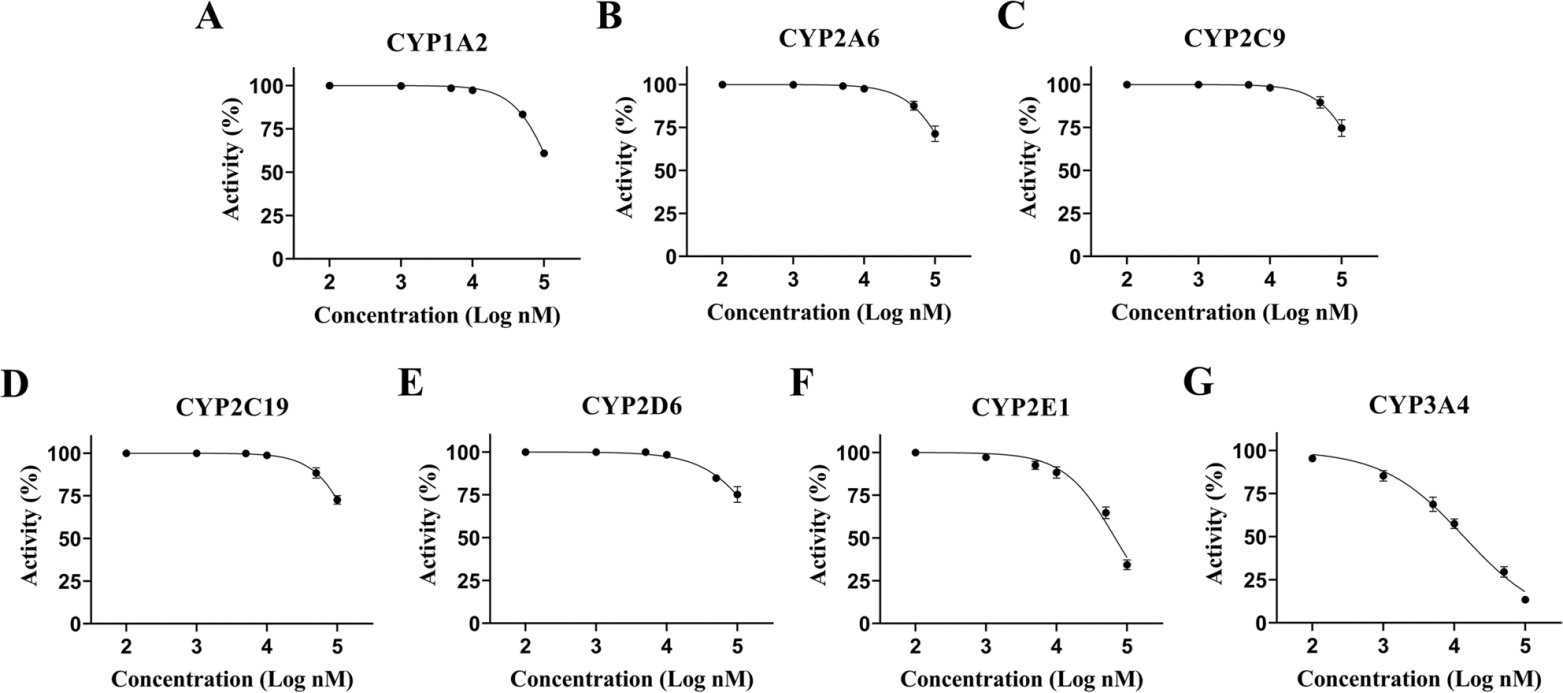
Inhibition curves of EDT on CYP1A2 (**A**), CYP2A6 (**B**), CYP2C9 (**C**), CYP2C19 (**D**), CYP2D6 (**E**), CYP2E1 (**F**) and CYP3A4 (**G**). The experiments were conducted in duplicate and the data are mean ± s.d.

The cytochrome P450 proteins are drug-metabolizing enzymes that involves in many catalytic reactions. Based on the fact that some pleuromutilin compounds, especially azamulin, displayed potent inhibition effect on CYP3A4^24^, we evaluated the inhibition potential of EDT on CYP450 enzymes using specific CYP probe substrates. The inhibitory effects of EDT on the seven major CYP450 enzymes were analyzed by determining IC_50_ values (Fig. 7). The results showed that EDT displayed a moderate inhibitory effect on CYP3A4 and a week inhibitory effect on CYP2E1, with IC_50_ of 9.25 and 88.51 μM, respectively. For CYP1A2, CYP2A6, CYP2C9, CYP2C19 and CYP2D6, EDT showed no inhibitory effect (IC_50_ > 100 μM).

### Microsomal stability

Finally, in vitro metabolic stability of EDT was quantified. A moderate metabolic stability in mouse and human liver microsomes was observed, with 30.12 and 43.61 min of half-life (*t*_1/2_), respectively. In the presence of mouse liver microsomes, EDT exhibited a high clearance (Cl_int_ = 46.02 μL/min/mg protein). While it resulted in a moderate clearance (Cl_int_ = 31.78 μL/min/mg protein) in human liver microsomes (Table 3).

**Table 3:** Metabolic stability of EDT in mouse or human liver microsomes.

Species	*t*_1/2_ (min)	Clint (μL/min/mg protein)
Mouse	30.12	46.02
Human	43.61	31.78

## Discussion

The novel pleuromutilin EDT has been proved to be a promising compound with higher antibacterial activities in vitro and in vivo against Gram-positive organisms, especially *S. aureus* and MRSA^21^. For further developing EDT to be a candidate veterinary drug, we performed its some antibacterial activities and toxicity investigation.

The MIC is an accepted and well used criterion for measuring the antibacterial potency of an inhibitor to organisms^25^, especially the clinical strains which might develop certain resistances after exposing to a variety of antibiotics for a long time. In present study, MIC values of EDT from 48 clinically isolated MRSA were range from 0.0313 to 0.0625 μg/mL, which were consistent with that from standard strains in our previously report^21^, indicating the robust inhibitory potency of EDT.

Drug resistance has always been an intractable problem and engenders the decreased availability of effective antibiotics. We stimulated MRSA continuously for 30 generations under the subinhibitory concentration (1/4 MIC) for simulating the environment of bacterial drug resistance. The result found that the MIC of tiamulin increased by 8 times, but only fourfold increase of MIC for EDT was observed. According to the criterion of resistance that was defined as an increase of fourfold more than the initial MIC^26^, EDT was just up to this criterion and more unsusceptible to drug resistance development than that of tiamulin in MRSA. However, after removing the drug, the bacteria returned to the normal susceptible level, indicating that the resistance of these compounds belongs to conditionally induced type.

The durations of the PAE of EDT were significantly prolonged than that of tiamulin with a concentration-dependent manner at the four tested concentrations. These results indicated that EDT may be administered with longer dosing intervals than currently employed tiamulin in veterinary clinical and without loss of efficacy^27^. We speculated that the relatively novel molecular structure of EDT formed more intermolecular hydrogen bonds with the bacterial 50S ribosome subunit than that of tiamulin, which could be reflected from the possible binding modes of EDT and tiamulin in the subsequent dock studies, and thus resulting in a prolonged period of bacterial recovery of ribosome protein synthesis ability.

For further investigating the higher antibacterial activity than that of tiamulin from molecular mechanism, we conducted the molecular docking and GFP inhibition assay. Molecular docking is helpful in predicting the potential ligand binding characteristics in the protein target^28,29^. Comparison of tiamulin, a π-π bond and more hydrogen-bonds in the predicted binding mode of EDT played an important role in increasing its affinity with PTC, and thus resulted in significantly higher antibacterial activity. Using *S. aureus* expressing GFP, EDT showed more noticeable effect on reducing GFP expression than that of tiamulin, directly demonstrating its higher antibacterial activity.

The safety profile of EDT was also evaluated. From a comparison of the cell viability data, we observed that EDT bearing substituted pyrimidine side chain displayed lower cytotoxicity than that of tiamulin. Furthermore, as deduced from Fig. 5, the CC_50_ of EDT to HepG2 and HEK293T should be > 200 μM (114.66 μg/mL), which were much higher than its MIC. In the acute toxicity study (2000 mg/kg/bw), no mortality or signs of toxicity at the macroscopic examination was found, indicating that LD_50_ value of EDT should be more than 2000 mg/kg body weight according to OECD Guidelines 423. These findings corroborate with our previous study, where a pleuromutilin compound with a pyrimidine moiety showed low acute toxicity with an approximate LD_50_ of 2973 mg/kg in female mice and 3891 mg/kg in male mice^30^.

Ames test is a rapid and effective screening method to evaluate the genetic toxicity of a chemical agent^31^. After validation of experimental strains, we conducted the preliminary experiment to determine the maximum dose of EDT to avoid the strains being inhibited. Five *S. typhimurium* strains and pKM101 with and without S9 metabolic activation were employed in the present study. The chemicals in positive control group significantly increased the counts of corresponding mutant strains than that in vehicle control groups. In the presence and absence of S9, exposure to five dosages of EDT did not increase the counts of revertant colonies significantly when compared to positive control group.

Cytochrome P450 enzymes catalyze the metabolism of a wide range of endogenous compounds, as well as xenobiotics including drugs, environmental pollutants and dietary products^32,33^. However, some chemicals inhibit CYP enzymes and trigger a series of adverse reactions^34^. One of detrimental properties of the pleuromutilin compounds is selective inhibition of CYP3A4, especially azamulin^24,35^. In this study, cocktail method was performed to evaluate the inhibitory effect of EDT on CYP1A2, CYP2A6, CYP2C9, CYP2C19, CYP2D6, CYP2E1 and CYP3A4. The results showed that EDT displayed a moderate inhibitory effect on CYP3A4 (IC_50_ was 9.25 μM), much weaker than that of azamulin and tiamulin which were reported to strongly inhibit CYP3A4 with IC_50_ of 0.24 μM and 1.60 μM, respectively^24,34^.

In drug discovery phase, accurate prediction of in vivo metabolic clearance using in vitro methods can greatly reduce the time-consuming and expensive animal studies^36^. Because the metabolites of EDT are still not known, we used the disappearance of EDT rather than the appearance of its metabolite to measure the metabolic stability. It was noted that this compound showed moderate stability against mouse and human liver microsomes, demonstrating that EDT may have an acceptable PK profile for further development.

## Materials and methods

### Chemicals

EDT (white powder) was synthesized in our laboratory according to the previous report^21^. The structure of this compound was determined by infrared spectroscopy (IR), nuclear magnetic resonance (NMR) and high resolution mass spectrometer (HRMS), and the purity was determined to be 99.6% by HPLC analysis (IR, 1H-NMR and 13C-NMR spectra were showed in Fig. S1). Tiamulin fumarate (purity of 98.5%) was purchased from Dr. Ehrenstorfer GmbH (Augsburg, Germany). All the other chemicals obtained from commercial sources were of analytical grade without further purification.

### Antibacterial activity against clinical isolates of MRSA

The tests against 48 clinical isolates of MRSA from dairy farms in 4 different provinces in China^37^ were conducted in triplicate, using broth serial-dilution method in 96-well plates according to CLSI reference methods^38^. Briefly, EDT and tiamulin fumarate used as referent drug were dissolved in 25% dimethyl sulfoxide (DMSO) to a solution with concentration of 64 μg/mL. The obtained 100 μL solutions were then added into the first column of the 96-well plate, and diluted to the tenth well with serial twofold dilutions. The last two wells were used as positive controls. The 100 μL bacterial suspension was diluted in MHB to give a final organism density of to 10^5^ ~ 10^6^ colony-forming unit (CFU) per 1 mL and added to all the wells, followed by incubating at 37 °C for 24 h. The MICs were determined as the minimum concentration at which a well showed no obvious bacterial growth by visual inspection. The results were expressed as an average of the MICs obtained from three independent experiments.

### The PAE test

The PAE of EDT against MRSA-337371 was determined using a standard viable counting method^27^. We selected 1 × , 2 × , 4 × and 8 × MICs of EDT and tiamulin (0.0313 and 0.5 μg/mL, respectively), to conduct PAE test on MRSA strains. The strains (1 × 10^5^ CFU/mL) were placed in MHB broth test tubes containing the above four concentrations of EDT and tiamulin, respectively, and cultured in a shaking bed at 37 °C for 1 h, followed by centrifuging for 10 min at 4000 r/min. The supernatants were discarded and the precipitations were dissolved with 2 mL normal saline to centrifuge. The same procedure was repeated three times. The final bacterial precipitates were taken and cultured in MHB broth with the initial concentration of 5.5 Log CFU/mL at 37 °C in a shaking bed. Samples were then taken at 0, 1, 2, 3, 4, 5, 6, 8, 12, 18 and 24 h to dilute 10–100,000 times and the 10 μL dilutions were evenly smear on MHA plate medium to culture at 37 °C for 48 h. Finally, the average numbers of colonies on the plates were obtained and the PAE values were calculated as hour using equation PAE = T − C, in which T was the time required for the increase of 1 log_10_ CFU/mL after the removal of drugs and C was the time required for the number of MRSA in the control tube to increase by 1 log_10_ CFU/mL. Experiments were performed in triplicate.

### Bacterial resistance study

We performed resistance induction tests on EDT and tiamulin which was used as a referent drug. MRSA-337371 was cultured for 16 h and diluted to 10^5^ CFU/mL with MHB broth medium. Subsequently, MRSA was inoculated onto fresh medium containing a subinhibitory concentration (1/4 MIC) of EDT and tiamulin, and cultured at 37 °C for 24 h. Finally, MRSA was cultured for 30 generations with the same procedure. At the 31st generation, drugs were removed until 40^th^ generation and MICs were determined in each generation.

### Docking procedures

The docking calculations were performed using the Schrödinger 2018 program package. The 50S ribosomal of *Deinococcus radiodurans* (*D. radiodurans*) in complex with tiamulin (PDB ID: 1XBP) prepared by use of the protein preparation wizard in software^39^, was simulated to contain all residues within a spherical cut of 20 Å from the PTC binding site. As a control, a redocking of tiamulin into the PTC model in which the drug was identical to its position in the X-ray structure. Docking was based on the Glide module^40^ in the Schrödinger 2018 program package in the XP (extra precision) mode. Flexibility was modeled by softening of the van der Waals radii with a scaling factor of 1.0 for the nonpolar parts of the receptor atoms and 0.9 for the ligand atoms. In both cases the charge cutoff was set to 0.2. Ranking of the docked ligands was based on the GlideScore (XP GScore).

### Green fluorescent protein (GFP) inhibition assay

The inhibition assay against GFP expression was performed according to our previous report^41^ with minor modification. Plasmids carried erythromycin resistant genes were labeled with GFP and electrotransformed into *S. aureus*. After screenings using erythromycin, *S. aureus* strain expressing GFP was then obtained and grown to mid-logarithmic phase, followed by collecting centrifugally and re-suspending with PBS buffer. The obtained suspensions were co-incubated with EDT and tiamulin with the final concentrations of 1 and 4 μM, respectively, for 4 h at 37 °C. The cells were subsequently collected by centrifugation to perform imaging using fluorescence microscopy after re-suspending with PBS buffer.

### Cytotoxicity assay

Cytotoxicity of EDT and tiamulin was studied against human hepatocellular carcinoma cells (HepG2) and human embryonic kidney 293T (HEK293T) cells following a protocol as published in our earlier reports^41,42^. Cells were counted and inoculated into 96-well plates with approximately10^5^ cells density for incubating 24 h at 37 °C. After removing medium, EDT and tiamulin (final concentrations of 0, 3.125, 6.25, 12.5, 25, 50 100 and 200 μM, respectively) in fresh medium (100 μL) were added into the plates and co-incubated for 6 h, followed by the addition of 10 μL of CCK-8 working solution to incubate for 4 h at 37 °C, the mediums were then tested at 450 nm using microplate reader (BioTek Instruments Inc., Winooski, USA). The same procedure was repeated three times. The percentages of cell viability were calculated by the formula: Cell viability (%) = [A_sample_ ‒ A_negative_]/[A_positve_ ‒ A_negative_] × 100%.

### Acute oral toxicity test

Adult specific pathogen free (SPF) female Wistar rats (4–6 weeks old) were purchased from Lanzhou Veterinary Research Institute, Chinese Academy of Agricultural Sciences. Animals were kept in clean stainless steel cages (3 rats per cage), free access to food and water and maintained under the 23 °C conditions with a constant 12 h light–dark cycle. Animals were acclimatized for at least 7 days before the experiment. The experimental procedures were conducted between 08:30 am and 17:30 PM in accordance with the ethical principles of animal research and approved by the Ethics Committee of the Laboratory Animal Center, Lanzhou Institute of Animal Science and Veterinary Medicine, Chinese Academy of Agricultural Sciences (No. 2022-005). The animal study was carried out in compliance with the ARRIVE guidelines.

Acute toxicity tests of EDT were performed according to OECD423 bulletin^23^. This method can measure drug roughly LD_50_ with fewer experimental animals. We selected 2000 mg/kg as the initial dose for first round treatment based on our previous report that a pleuromutilin compound with pyrimidine side chain displayed lower acute oral toxicity in mice^30^. Six animals were randomly separated into one treatment group (n = 3) and control group (n = 3). Rats in treatment group were orally administered with a single dose of 2000 mg/kg of EDT dissolved in vehicle (corn oil: DMSO = 4:1) in a volume of 10 mL/kg body weight (b.w.) by gavage. Animals in the control group only received 10 mL/kg vehicle. All animals were observed individually in open field at 10, 30, 60, 120 and 240 min, and once a day for 14 consecutive days. After first round treatment, observations were focused on mortality, behavioural symptoms and changes in skin, eyes and fur. Individual body weight was recorded at the starting of protocol and at the end of protocol. Because no animal died during the first 7 days after treatment, we conduct the second round treatment with the same procedure to test the acute oral toxicity on three additional female rats. On the 14^th^ day, all the surviving animals were euthanized with ketamine hydrochloride 70 mg/kg (i.p.) and their hearts, livers, spleens, lungs, kidneys, uterus and ovaries were individually observed for overt pathology and removed for relative weight calculation (organ/b.w. × 100).

### Ames test

Before the test, *Salmonella Typhimurium* (*S. Typhimurium*) TA97a, TA98, TA100, TA102, TA1535 and *Escherichia coli* WP2uvrA pKM101 (pKM101) were first checked for their genetic integrity, including biotin dependence, *rfa* marker (crystal violet), biotin, histidine dependence and presence of the plasmid pKM101 (ampicillin resistance) or pAQ1 (tetracycline resistance). The results showed that all the strains were normal and suitable for the further test (data were showed in Table S3). The maximum concentration of EDT was also screened using a preliminary experiment. The results showed that 5000 μg/plate dose of EDT inhibited the test strains, but 2000 μg/plate dose has not toxic. Therefore, we selected 2000 μg/plate as the maximum dose for the further tests.

The test was performed with 20,000, 4000, 800, 160 and 32 μg/mL of EDT dissolved in DMSO. The obtained serial dilutions (0.1 mL) were diluted with 1.9 mL melted top agar to the desired concentrations (1000, 200, 40, 8 and 1.6 μg/mL). DMSO (0.1 mL) was directly incorporated into 1.9 mL melted top agar as the vehicle control. Four different positive control chemicals, including 2-aminofluorene (10 μg/plate), dexon (50 μg/plate), methyl sulfonate (1μL/plate), 2-aminoanthracene (10 μg/plate) and sodium azide (1.5 μg/plate) were used for each tested strain with or without S9. Each strain (0.1 mL, 6–7 log_10_ CFU/mL) was added into 2 mL of top agar in which comprised 0.5 mL S9 (10%), serial concentrations of EDT (2000, 400, 80, 16 and 3.2 μg/plate), DMSO or control chemicals for testing with the S9. For testing without S9, 0.5 mL phosphate buffer saline (PBS, pH 7.4) replaced the S9 mix. Then, the obtained top agar was melted, poured onto the surface of nutrient broth and swirled to distribute the top agar to all surfaces of medium, followed by incubation at 37 °C for 48 h. The resultant colonies were counted manually as the number of revertant colonies per plate. The experiments were performed in triplicate and the results were expressed as the average ± SD.

### Inhibitory effects on cytochrome P450

Inhibition effects of EDT on cytochrome P450 were evaluated with rat liver microsomes in vitro^43^. Briefly, 12.8 mg EDT was dissolved in methanol and stored at 4 °C as reserve solution. A variety of specific probe substrate (1 μL), EDT solution (1 μL), incubation mixture (193 μL) in which contained glucose-6-phosphate, glucose-6-phosphate dehydrogenase, potassium phosphate buffer, magnesium chloride buffer and PBS buffer, were added into 5 μL liver microsomes to start the reaction at 37 °C. The final concentration of EDT in each group was 0.1, 1, 5, 10, 50, 100 μM. After 30 min, the reaction was terminated by adding the same volume of pre-cooled acetonitrile into the incubation system. After centrifugation for 10 min (8000×*g* at 4 °C), 10 μL supernatant was removed to analyze the metabolites of specific probe substrate by HPLC. The effects of EDT on CYP450 subtypes were evaluated using formula as follow: (1 − C_n_/C_0_) × 100%, where C_n_ was substrate metabolite of different concentration of EDT, and C_0_ was metabolite of blank group.

### Microsomal stability assay

Mouse and human liver microsomes were thawed slowly on ice. A total of 5 mg/mL of microsomes, 2 μL of a 100 μM solution of EDT and 183 μL of 100 mM phosphate buffer were incubated 5 min at 37 °C in a water bath. Reactions were initiated using 10 μL of 20 mM NADPH. Samples were incubated in three replicates at 37 °C under gentle agitation at 150 rpm. At 0, 5, 15, 30, and 60 min, reactions were terminated by the addition of 180 μL of acetonitrile. After centrifugation (4000 rpm, 10 min), samples were then analyzed by liquid chromatography/tandem mass spectrometry (LC–MS/MS). Peak areas of the respective time point of the compound were normalized to the peak area at time point 0 min. Then half-life was calculated using linear regression from log percentage (*t*_1/2_ = − 0.693/k). Cl_int_ [μL/min/mg protein] was calculated using the formula: Cl_int_ = 0.693/(0.0005 mg/μL × *t*_1/2_)^36,44^.

### Statistical analysis

Results of cell viabilities and the body weights of rats in acute oral toxicity were expressed as mean ± standard deviation (SD). Differences between groups were determined by a one-way analysis of variance (ANOVA), followed by Dunnett's post-hoc tests using IBM SPSS Statistics for Windows version 24.0^45^. Statistical significant differences were defined as a *p* < 0.05 and the extremely significant differences were defined as a *p* < 0.01.

## Conclusions

In summary, pleuromutilin compound EDT exhibited significant antibacterial activity and potent safety profile. In in vitro antibacterial assays, EDT displayed higher bactericidal activity against clinical isolates of MRSA, better post-antibiotic response and relatively difficult to develop drug resistance when compared to that of tiamulin. Docking model and inhibition study on the expression of GFP were further confirmed the higher antibacterial activities of EDT. Safety evaluation revealed the negligible cytotoxicity, low acute toxicity in rats, not mutagenic response under the present Ames test and weak inhibitory potential on CYP3A4. The acceptable microsomal stability further provides the basis for further developing EDT to a potential anti-bacterial clinical candidate.

### Supplementary Information

Supplementary Information.

## Data Availability

The original contributions presented in the study are included in the article/supplementary material.
