# Supplementary material for: Anti-methicillin-resistant Staphylococcus aureus activity and safety evaluation of 14-O-[(5-ethoxycarbonyl-4,6-dimethylpyrimidine-2-yl) thioacetyl] mutilin (EDT)
Source: Sci Rep. 2023 Sep 14;13:15267. doi: 10.1038/s41598-023-42621-0 (PMC10502144; doi:10.1038/s41598-023-42621-0)
Supplement: Supplementary file 1 — Supplementary Information. [file 41598_2023_42621_MOESM1_ESM.pdf]

Anti-methicillin-resistant *Staphylococcus aureus* activity and safety evaluation of EDT: a novel pleuromutilin candidate

Yuhang Zhou[a], Yunpeng Yi[b], Jing Yang[c], Hongjuan Zhang[a], Qinqin Liu[a], Shengyi Wang[a], Wanxia Pu[a], Ruofeng Shang[a*]

[a]*Key Laboratory of New Animal Drug Project, Gansu Province/Key Laboratory of Veterinary Pharmaceutical Development, Ministry of Agriculture and Rural Affairs/ Lanzhou Institute of Husbandry and Pharmaceutical Sciences of CAAS, Lanzhou, 730050, China.*

[b]*Shandong Provincial Animal and Poultry Green Health Products Creation Engineering Laboratory, Institute of Poultry Science, Shandong Academy of Agricultural Science, Jinan, 250023, China.*

[c]*Gansu Analysis and Research Center, Lanzhou, 730000, China.*

Corresponding author: Tel.: +86 931 2115253; fax: +86 931 2115951. E-mail address: shangrf1974@163.com (Ruofeng Shang)

Author: Tel.: +86 18860362851; E-mail address: zhouyuhangcaas@163.com (Yuhang Zhou) and yiyp@foxmail.com (Yunpeng Yi). Yuhang Zhou and Yunpeng Yi contributed equally to this work.

Address: Lanzhou Institute of Husbandry and Pharmaceutical Sciences of CAAS, No. 335, Qilihe District, Lanzhou, 730050, PR China.

Supplemental Data

Table of Contents

Figure S1. IR, ^{1}H-NMR and ^{13}C-NMR spectra of EDT……1

Figure S2. Concentrations of the strains taken at different times after removing drugs in PAE test……3

Table S1. MIC values of EDT and tiamulin fumarate against clinical isolates of MRSA……4

Table S2. MIC values of EDT and tiamulin fumarate in the resistance development study……5

Table S3. Identification results of all the examined strains in Ames test……6

A

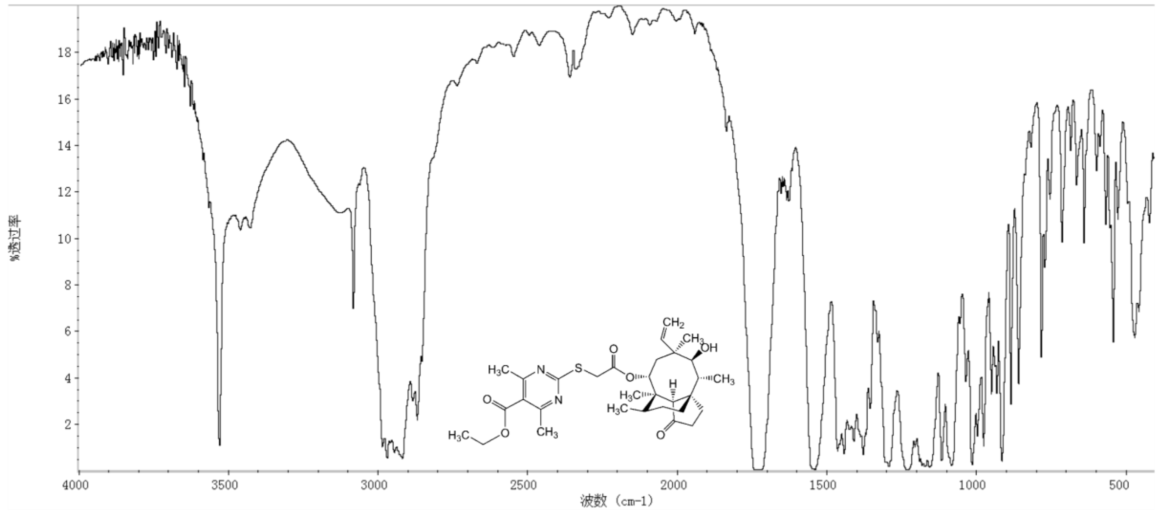

B

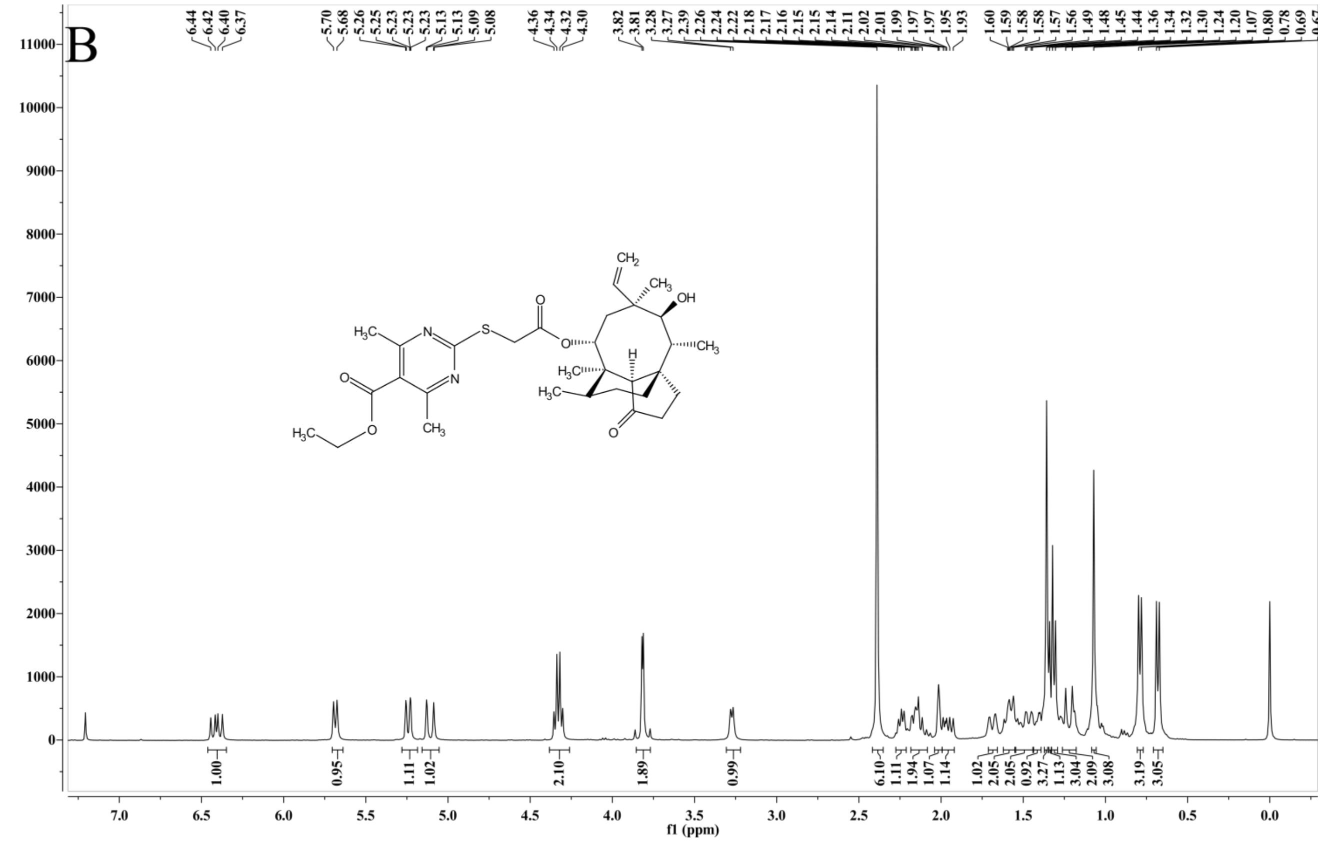

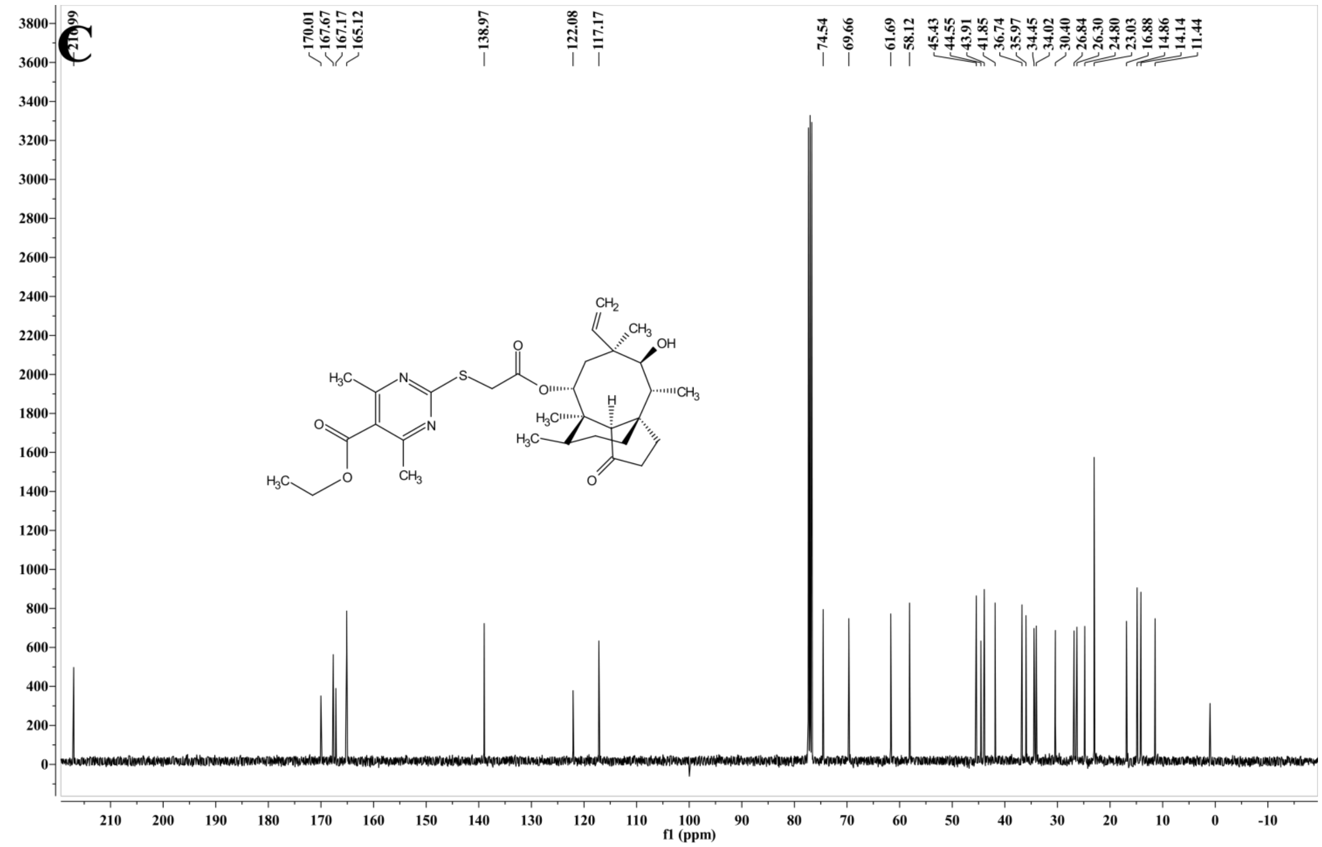

Figure S1. IR (A), ^{1}H-NMR (B) and ^{13}C-NMR (C) spectra of EDT

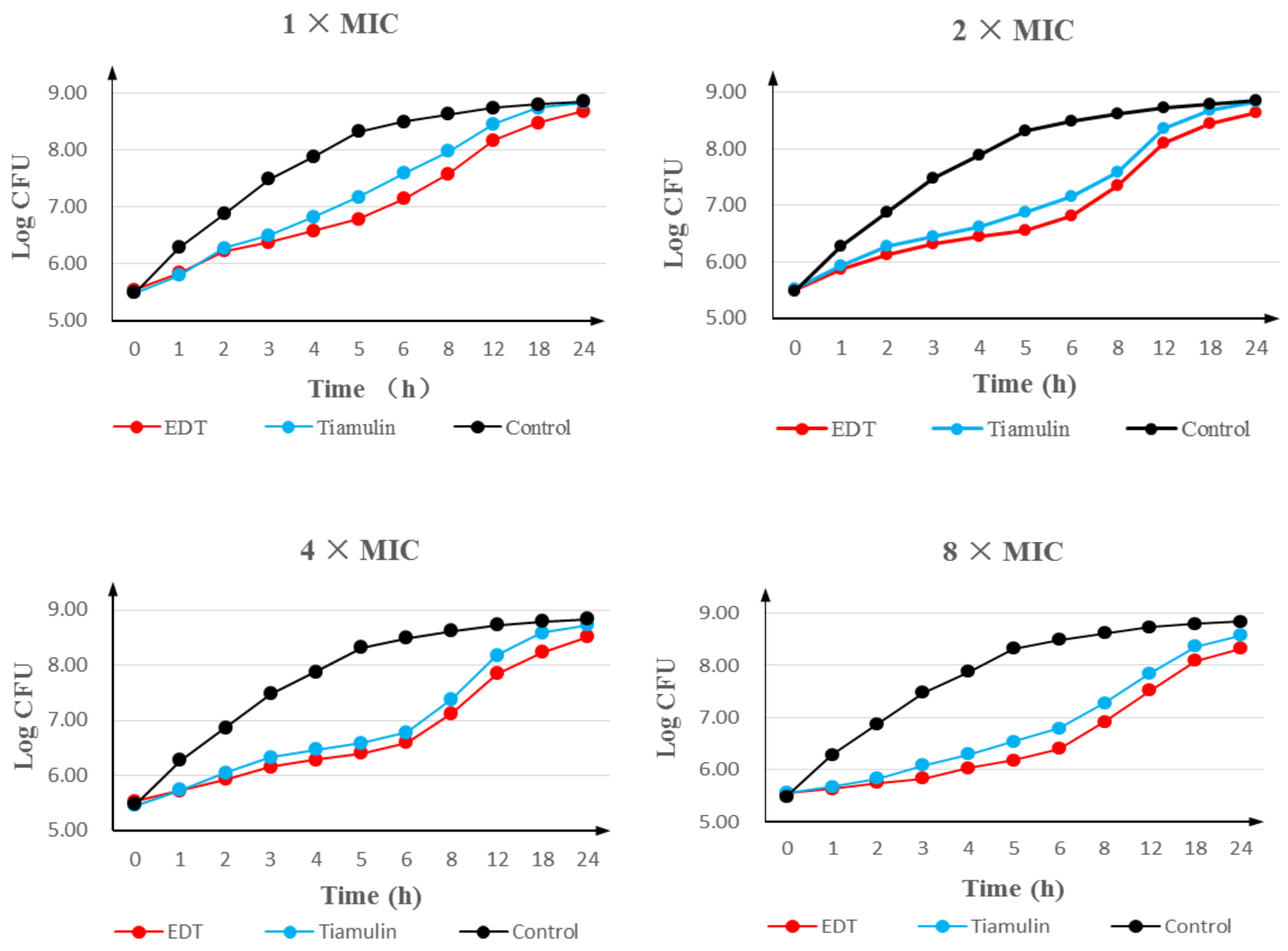

Figure S2. Concentrations of the strains taken at different times after removing drugs in PAE test

Table S1. MIC values of EDT and tiamulin fumarate against clinical isolates of MRSA.

Stain[a]	MIC (μg/mL)		Stain[a]	MIC (μg/mL)	
	EDT	Tiamulin		EDT	Tiamulin
QY4	0.0313	0.5	ZY6	0.0313	1
QY5	0.0313	0.5	SH10	0.0313	0.5
SH3	0.0313	0.5	SH11	0.0313	0.5
SH4	0.0313	0.5	SH12	0.0313	0.5
SH6	0.0313	0.5	SH13	0.0313	0.5
SH7	0.0625	0.5	SH14	0.0313	0.125
SX5	0.0313	0.5	QY10	0.0625	0.5
HG1	0.0313	0.25	QY6	0.0625	0.5
HG2	0.0313	0.5	HG4	0.0313	0.5
SX16	0.0625	1	HG5	0.0313	0.5
SX17	0.0313	0.5	HG6	0.0313	0.5
SX18	0.0313	0.5	SH8	0.0313	0.25
CX1	0.0313	0.5	SH9	0.0313	0.5
CX2	0.0313	0.5	SX8	0.0313	0.5
QY8	0.0313	0.5	SX9	0.0313	0.5
CX5	0.0313	0.5	SX10	0.0313	0.5
CX6	0.0313	0.5	SH15	0.0313	0.5
CX7	0.0625	0.5	HG7	0.0625	0.5
ZY4	0.0313	0.5	ZY1	0.0313	0.5
SX6	0.0313	1	ZY2	0.0313	0.5
SX7	0.0313	0.5	ZY3	0.0313	0.25
HG3	0.0313	0.5	ZY7	0.0313	0.5
CX9	0.125	1	ZY8	0.0625	1
ZY5	0.0313	0.5	ZY9	0.0313	0.25

[a]Strains were collected from dairy farms located in 4 different provinces in China.

Table S2. MIC values of EDT and tiamulin fumarate in the resistance development study.

day	MIC (μg/mL)		day	MIC (μg/mL)		day	MIC (μg/mL)	
	EDT	Tiamulin		EDT	Tiamulin		EDT	Tiamulun
1	0.0313	0.5	15	0.0313	1	29	0.125	4
2	0.0313	0.5	16	0.0313	2	30	0.125	4
3	0.0313	0.5	17	0.0625	2	31	0.125	4
4	0.0313	0.5	18	0.0625	2	32	0.125	4
5	0.0313	0.5	19	0.0625	2	33	0.0625	2
6	0.0313	0.5	20	0.125	4	34	0.0625	2
7	0.0313	1	21	0.125	2	35	0.0313	2
8	0.0313	1	22	0.125	4	36	0.0313	1
9	0.0313	1	23	0.125	4	37	0.0313	1
10	0.0313	1	24	0.125	4	38	0.0313	0.5
11	0.0313	1	25	0.125	4	39	0.0313	0.5
12	0.0625	1	26	0.125	4	40	0.0313	0.5
13	0.0625	1	27	0.125	4			
14	0.0313	2	28	0.125	4			

[a]Strains were collected from dairy farms located in Gansu province, China.

[b]Multilocus sequence typing (MLST) of microbe.

Table S3. Identification results of all the examined strains in Ames test.

Experimental strains	Histidine defect	Lipopolysaccharide barrier deficiency	R factor (ampicillin)	tetracycline resistance	uvrB fixes defects
TA97a	＋	＋	＋	－	＋
TA98	＋	＋	＋	－	＋
TA100	＋	＋	＋	－	＋
pKM101	No	No	＋	－	＋
TA102	＋	＋	＋	＋	－
TA1535	＋	＋	－	－	＋

Note: "＋" indicates a positive result, "－" indicates a negative result, and "No" indicates that no verification is required.
